# Stressors and bathroom behaviors associated with urinary incontinence in nurses working during the COVID-19 pandemic: A mixed-methods study

**DOI:** 10.1097/nmg.0000000000000124

**Published:** 2024-04-29

**Authors:** Elissa Allen, Kelly Ackerson

**Affiliations:** At Bronson School of Nursing, Western Michigan University in Kalamazoo, Mich., **Elissa Allen** is an assistant professor and MSN coordinator, and **Kelly Ackerson** is a professor emeritus.

## Abstract

An inability to take breaks contributed to premature and delayed voiding with a high prevalence of stress and urge incontinence. Nurse leaders can implement policies to promote healthy toileting behaviors.

**Figure:**
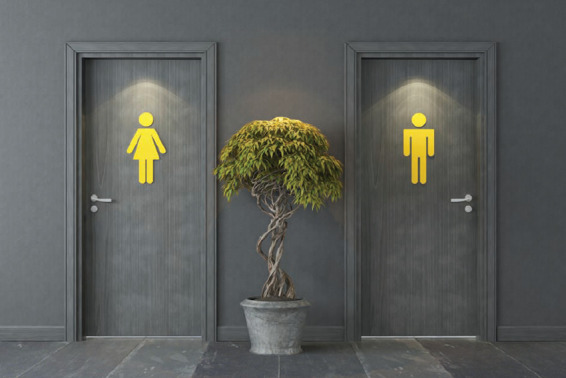
No caption available.

Lower urinary tract symptoms (LUTS) are highly prevalent in women in the US.^1^ LUTS is an umbrella term and includes a combination of urinary symptoms such as urinary incontinence, urinary tract infection (UTI), and overactive bladder. RNs have a disproportionately higher prevalence of LUTS, with over 60% of nurses reporting at least one symptom.^1^,^2^ Two highly prevalent forms of LUTS are stress urinary incontinence (SUI) and urinary urge incontinence (UUI). The high prevalence of LUTS in nurses is significant, as research has shown that urinary incontinence reduces quality of life and creates a large financial burden, both individually and for the US as a whole, and that nurses are particularly affected.^2^,^3^

SUI and UUI are caused in part by unhealthy bladder behaviors, including straining while voiding, voiding prematurely, and delayed voiding. Straining when voiding entails using the abdominal muscles to push the urine out of the bladder to speed up the voiding process. Voiding in advance of the biological urge to do so is considered premature voiding. When one is delaying voiding, they're ignoring the urges to void and instead “hold urine.” These behaviors have been shown to contribute to LUTS development over time.^4^

One of the major contributing risk factors for LUTS development in nurses is delayed voiding. Voiding delays weaken the pelvic floor muscles, which help maintain continence. Nurses may prioritize caring for patient needs over the biological urge to void, thus delaying access to a bathroom to empty their bladder. An additional factor that can affect the bladder is psychological stress, which is a common finding in the work environment of many nurses.^5^

During the COVID-19 pandemic, there may have been an additional risk to nurses' bladder health, as they cared for increased numbers of high-acuity patients and experienced higher levels of stress.^6^,^7^ As occupational stress (OS) increases for nurses, they may experience strained, premature, and/or delayed voiding. It's unknown how the COVID-19 pandemic affected nurses' ability to access the bathroom during working hours and how their toileting habits were affected.

This study investigated: 1) toileting behaviors of nurses; 2) associations that different toileting behaviors have with OS and SUI and UUI symptoms; and 3) the stressors that nurses experienced during the COVID-19 pandemic that influenced their bathroom behaviors at work.

## Methods

### Participant recruitment

This study was approved by the institutional review board (IRB) for human participants at the researchers' home institution.

**Phase 1**. RNs age 18 years and older, who were assigned female at birth, spoke and read English, and worked in a hospital anywhere in the US during the COVID-19 pandemic were included. Recruitment occurred through online advertising on Facebook and Instagram over a 12-week timespan. In addition, to broaden recruitment, the primary investigator (PI) emailed the study advertisement to colleagues and nurse managers in different states requesting their assistance in sharing the advertisement. A drawing for one of 10 $50 Amazon gift cards was offered at the end of the survey. No personal information collected for the drawing was linked to participants' data.

**Phase 2**. Survey respondents interested in participating in a one-on-one, semistructured virtual interview submitted their name and contact information at the end of the survey (*Phase 1*) and were offered a $50 Amazon gift card as compensation for their time. The purpose of the interviews was to elicit additional information related to bathroom behavior while working during the COVID-19 pandemic. Participant recruitment was planned until data saturation was reached.

#### Informed consent process

**Phase 1**. Potential participants who clicked on the survey link were taken to the informed consent. If they self-identified as meeting the inclusion criteria and interested in participating, clicking on the link gave them direct access to the survey, and entering the survey implied consent. Participants were informed they could stop participating at any time by simply exiting the survey and no data would be collected.

**Phase 2**. Participants from *Phase 1* who provided contact information to participate in the one-on-one interviews were sent an email invitation with informed consent attached. They were asked to contact the PI if still interested in setting up an interview. At the scheduled meeting time, informed consent was reviewed and questions were answered before the interview, which immediately followed.

### Phase 1: Data collection

The PI sent a 70-item, electronic, Qualtrics survey to nurses in February 2021 through social media and community connections. Included in the survey were demographics and three survey instruments. Length of time to complete the survey averaged 15 to 20 minutes.

**Survey instruments**. The investigators used the Michigan Incontinence Symptom Index (M-ISI) to evaluate SUI (leakage of urine during physical activity) and UUI symptoms that participants experienced during the previous month.^8^ The M-ISI can be used to determine clinically relevant incontinence. It's a 10-item scale with two domains (M-ISI Domain, 8 items; and Bother Domain, 2 items) that uses a 5-point scale ranging from 0 (never) to 4 (most or all of the time). The M-ISI domain includes three subdomains: SUI (Items 1 through 3), UUI (Items 4 through 6), and pad use (Items 7 through 8). The study focused on the subdomains of SUI (Items 1 through 3, with a possible sum of 0 to 12) and UUI (Items 4 through 6, with a possible sum of 0 to 12). The clinically relevant incontinence threshold for SUI is greater than or equal to 3, and the threshold for UUI is greater than or equal to 5.^9^ This scale is reliable and valid for use with adult women. In this study, internal consistency was 0.92.

The Nurses Occupational Stressors Scale (NOSS) was used to investigate OS.^10^ This instrument includes 21 items with nine subscales. Responses use the Likert style of 1 (strongly disagree) to 4 (strongly agree) with a range of scores from 21 to 84, with higher scores indicating greater perceived OS. NOSS has high concurrent and construct validity with subscale correlation coefficients for test-retest reliability ranging from 0.71 to 0.83.^10^ For this study, the NOSS internal consistency was 0.88.

To investigate toileting behavior, the English version of the Toileting Behaviors-Women's Elimination Behaviors (TBWEB) instrument was used.^11^ This scale includes 26 items with seven subscales with high reliability and validity in samples of US nurses.^11^ Internal consistency ranged from 0.70 to 0.88 and was 0.83 for this study.

**Data analysis**. Analysis methods included descriptive and inferential statistics calculated via SPSS v.27. To address aim 2, logistic regressions were used and adjusted for demographic and influential factors.

### Phase 2: Data collection

Interviews occurred from August 2021 through January 2022, after completion of the online survey. The PI and co-investigator developed semistructured interview guide questions based on Phase I findings to elicit additional information related to bathroom use at work during the COVID-19 pandemic. Interviews were audio-recorded and transcribed verbatim. Deidentified audio files from the interviews were sent to a transcription company. Before qualitative analysis, the research team verified transcript accuracy.

**Data analysis**. Qualitative data were analyzed using thematic analysis.^12^ Additionally, field notes and memos were used to support credibility. To aid in qualitative coding, AtlasTI v.22.0.6.0 was used. Each investigator independently analyzed the data before coming together to discuss and agree on identified themes.

## Results

A total of 602 questionnaires were returned, of which 334 were complete and used for analysis after eliminating surveys that had item nonresponses (>5% responses missing) and any survey submitted that took 5 minutes or less to complete. Many participants were between the ages of 18 and 30 (47.9%), Black (47%), and married (64.7%). More than 56% of participants had worked as a nurse for 3 to 6 years, and over 50% of the participants reported a history of UTIs. Many participants reported never being pregnant (42.5%), and of those who reported at least one pregnancy (37.1%), 37.4% delivered vaginally. The presence of LUTS in the previous month was reported in 82.3% of the sample, and of those individuals, 82% had SUI and 55.6% had UUI. Most of the sample offered clinical relevance for LUTS, predominantly SUI (see Table 1).

**Table 1:** Participant demographics

Variables	n (%) or Mean (SD)
**Age, y**	
18-30	160 (47.9%)
31-40	148 (44.3%)
41-50	16 (4.8%)
51-60	7 (2.1%)
61-70	3 (0.9%)
**Gender identity**	
Agender	34 (10.2%)
Gender nonconforming	16 (4.8%)
Genderqueer	13 (3.9%)
Transgender	1 (0.3%)
Woman	229 (68.5%)
Prefer not to share	21 (6.3%)
**Race**	
Black	157 (47%)
Native American or Alaska Native	63 (18.9%)
Asian	8 (2.1%)
White	64 (19.2%)
Native Hawaiian or other Pacific Islander	9 (2.7%)
Prefer not to say	32 (9.6%)
Other	2 (0.6%)
**Marital status**	
Married	216 (64.7%)
Widowed	16 (4.8%)
Divorced	20 (6%)
Separated	10 (3.0%)
Never married	73 (21.6%)
**Income**	
<$20,000	11 (3.3%)
21,000-39,999	119 (35.5%)
40,000-59,999	116 (34.6%)
60,000-79,999	41 (12.2%)
80,000-99,999	13 (3.9%)
100,000-149,999	22 (6.6%)
150,000-199,999	8 (2.4%)
200,000+	5 (1.5%)
**Number of pregnancies**	
0	142 (42.5%)
1	124 (37.1%)
2	48 (14.4%)
3	11 (3.3%)
4+	8 (2.4%)
**Number of vaginal births**	
0	157 (47%)
1	125 (37.4%)
2	42 (12.5%)
3	9 (2.7%)
4+	1 (0.3%)
**Number of cesarean sections**	
0	258 (77.2%)
1	59 (17.7%)
2	14 (4.2%)
3	2 (0.6%)
4+	1 (0.3%)
**History of UTIs**	
Yes	182 (54.5%)
No	147 (44%)
**Typical fluid intake (8 oz glasses per day)**	
1-2	29 (8.7%)
3-4	134 (40.1%)
5-6	117 (35%)
7-8	47 (14.1%)
9-12	4 (1.2%)
12+	3 (0.9%)
**Highest level of education**	
Below a bachelor's degree	115 (34.3%)
Bachelor's degree or higher	219 (65.4%)
**Years worked as RN**	
0-2	77 (23.1%)
3-6	188 (56.2%)
7-10	47 (14.1%)
11-20	12 (3.6%)
21+	10 (3%)
**Professional title**	
Registered nurse	192 (57.3%)
Charge nurse	54 (16.1%)
Unit coordinator	22 (6.6%)
Nurse practitioner	21 (6.3%)
Unit director	11 (3.3%)
Clinical nurse specialist	10 (3.0%)
Nurse midwife	21 (6.3%)
Nurse anesthetist	1 (0.3%)
Other	3 (0.9%)
**M-ISI**	
SUI ≥3	4.30 (2.13)
UUI ≥5	4.66 (2.18)
**SUI**	
<3	59 (17.7%)
≥3	273 (82%)
**UUI**	
<5	148 (44.4%)
≥5	185 (55.6%)
**Occupational stress (NOSS)**	53.53 (8.99)

Table 2 provides information on the top 10 occupational stressors. The number one occupational stressor affecting nurses working during the pandemic was having to adapt their work schedule around the family, followed closely by having insufficient time to offer mental health care to patients. Other top occupational stressors connected to bladder health included an inability to take an uninterrupted 30-minute mealtime break or to take time to fulfill personal needs such as drinking water and/or taking toilet breaks.

**Table 2:** Top 10 occupational stressors rated from 1 (strongly disagree) to 4 (strongly agree)

Occupational stressor	Mean (SD)
I have to adapt my schedule for family activities/outings to accommodate my work responsibilities.	2.71 (.77)
I have insufficient time to offer mental health care to patients during working hours.	2.69 (.78)
I can't take an uninterrupted 30-minute mealtime break.	2.68 (.77)
I have no time to fulfill my personal needs (for example, drink water and/or take toilet breaks).	2.68 (.76)
It upsets me if patients' conditions don't improve.	2.68 (.71)
I feel stressed that my patients might have contagious diseases such as COVID-19.	2.63 (.79)
The burden of work makes it difficult for me to undertake my personal chores and/or engage in hobbies.	2.63 (.80)
The burden of work affects my personal life.	2.62 (.74)
I can't leave during a shift even if I'm sick or in pain.	2.60 (.75)
I'm usually the one who has to transport patients or equipment.	2.60 (.80)

Delayed voiding was the most common unhealthy toileting behavior, reported by over 43% of nurses. Outside of the home, more than one-third of the nurses felt that they waited too long to either void or empty their bladder until they felt they couldn't hold their urine any longer. Place preference for voiding was also an issue, with more than 45% of the nurses worrying about how clean public toilets were and more than one-third of nurses preferring to empty their bladders before leaving home (46.6%). Additionally, 35% of the nurses reported premature voiding at home, indicating that they emptied their bladder even if they didn't feel the urge. (See Tables 3 and 4.)

**Table 3:** Toileting positions

	Never/rarely n (%)	Sometimesn (%)	Often/alwaysn (%)
**At home**			
Sit on the toilet seat to urinate	25 (7.5)	59 (17.7)	246 (73.6)
Crouch (hover) over the toilet when I urinate	158 (62.3)	88 (26.3)	37 (11.1)
Squat on the toilet seat to urinate	226 (67.7)	69 (20.7)	37 (2.1)
Stand over the toilet with my feet on each side of the toilet bowl, knees bent, facing toward or away from the toilet to urinate	290 (86.9)	27 (8.1)	15 (3.9)
**Away from home**			
Sit on the toilet seat to urinate	58 (17.4)	63 (18.9)	211 (36.1)
Crouch (hover) over the toilet when I urinate	167 (50)	112 (33.5)	53 (15.9)
Squat on the toilet seat to urinate	225 (67.3)	61 (18.3)	45 (13.5)
Stand over the toilet with my feet on each side of the toilet bowl, knees bent, facing toward or away from the toilet to urinate	278 (83.3)	45 (13.5)	8 (2.4)

**Table 4:** Toileting behaviors

	Never/rarely n (%)	Sometimes n (%)	Often/always n (%)
**Delay voiding**			
Delay emptying bladder when busy	47 (14.1)	137 (41)	146 (43.7)
Wait to empty bladder until feel you can't hold urine	65 (19.5)	151 (45.2)	117 (35)
Wait too long	79 (23.7)	136 (40.7)	119 (35.6)
**Straining to void (strain/tighten abdominal muscles)**			
Don't think about emptying bladder completely	94 (28.2)	125 (37.4)	112 (33.3)
Push down to begin urinating	84 (25.2)	143 (42.8)	107 (33)
Push down to keep urine flowing	67 (20.1)	154 (46.1)	111 (33.2)
Push down in order to empty bladder	86 (25.8)	138 (41.3)	109 (32.6)
Push down to empty faster	63 (18.9)	155 (46.4)	115 (34.4)
**Premature voiding**			
When at home, empty bladder even if you don't feel the need to urinate	103 (30.9)	114 (34.1)	117 (35)
When away from home, empty bladder even if you don't feel the need to urinate	114 (34.2)	131 (39.2)	86 (25.8)
When at someone else's home, empty bladder even when you don't feel the need to urinate	140 (41.9)	121 (36.2)	73 (21.9)
When in public places, empty bladder even when you don't feel the need to urinate	135 (40.5)	117 (35)	79 (23.7)
Empty bladder with little or no need to urinate “just in case”	86 (25.8)	152 (45.5)	95 (28.5)
**Place preference for voiding**			
Worry about how clean public toilets are	73 (21.9)	109 (32.6)	152 (45.5)
Avoid using public toilets	80 (24.0)	141 (42.2)	112 (33.5)
Empty bladder before leaving home	59 (17.7)	115 (34.4)	159 (47.6)
Hold urine until getting home	79 (23.7)	142 (42.5)	112 (33.5)

In Table 5, the results of the logistic regression for each toileting behavior subscale indicate that OS didn't exhibit an association with either SUI or UUI after controlling for toileting behaviors and other influencing variables. However, premature voiding and straining to void were significantly associated with SUI (odds ratio = 1.222, *P* ≤ .001; odds ratio = 1.232, *P* = .007, respectively), and straining to void was significantly associated with UUI (odds ratio = 1.173, *P* = .013). Place preference had a negative association with UUI (odds ratio = 0.832, *P* = .001).

**Table 5:** Association of OS and toileting behaviors with SUI and UUI

	Association with SUI			Association with UUI		
	OR	SE	*P* value	OR	SE	*P* value
Occupational stress	.992	.023	.732	.999	.018	.964
Place preference for voiding	.976	.073	.742	.832	.056	.001
Premature voiding	1.222	.057	<.001	1.055	.044	.221
Delay voiding	.872	.113	.226	1.001	.087	.988
Straining voiding	1.232	.077	.007	1.173	.064	.013
Normal toileting behavior	.829	.153	.222	.954	.114	.677
Age	.980	.260	.937	1.347	.189	.115
Number of vaginal births	1.455	.263	.154	.834	.193	.348
Fluid intake	.802	.185	.233	.865	.149	.331
History of UTIs	2.335	.385	.027	2.635	.286	<.001
Years worked as an RN	1.228	.266	.441	1.114	.207	.601

Key: OR, odds ratio; SE, standard error

### Qualitative findings

The nurses who participated in phase 2 of this study (N = 10) were managers (n = 2) and floor nurses (n = 8), and three of the eight were travel nurses. Many of the participants worked 12-hour shifts in medical-surgical units, ICUs, and EDs. All but one participant cared for patients with COVID-19, and all participants indicated that adequate hydration and use of the bathroom were affected when working during the pandemic. Themes emerged surrounding OS, bathroom behaviors, and variations in staffing during waves of increased COVID-19 infection.

### Occupational stress and bathroom behaviors

Participants reported that during the COVID-19 pandemic, they couldn't leave their patients and take bathroom breaks, which contributed to an increase in straining during voiding, premature voiding, and delayed voiding. When describing delayed voiding, one participant stated: “If I had to go pee, it wasn't happening. I couldn't just head out of the room. I kind of had to hold it. I wouldn't want to get up [from charting] to do anything, to go to the bathroom, so I would try to hold it.”

In addition to delayed voiding due to busy patient loads, participants also reported using the bathroom prematurely. One participant commented, “You see a window of opportunity, and you grab it.” Using the bathroom “just because” was common, especially in the subsequent waves of the pandemic. A new nurse indicated that before becoming a nurse, they never “prematurely” went to the bathroom unless they had to and that the bathroom habits they developed during the pandemic affected their bathroom habits outside of the work environment: “I have never done that before until I became a nurse. I'm sure a lot of it has to do with the pandemic because most of my nursing experience is in the pandemic. Even outside of work, I do that now too.”

### Variability in staffing during COVID-19 waves

During the first wave of the pandemic (late 2019 through early 2020), participants indicated that there was sufficient staff to cover bathroom breaks. However, during the second wave of the pandemic (late 2020 through early 2021), participants reported less staff, and they were busier, restricting bathroom breaks: “If I knew when I went to the bathroom, if I had a floating person constantly where I was like, ‘Hey, I'm running [to] pee. Can you go start this I.V. and draw blood on that patient and keep an eye on this patient's blood pressure?’ I would feel more comfortable going to the bathroom, and taking 15 minutes, and going when I feel like I have to go, but the fact that you're so constantly busy, and if you go to the bathroom, that's the 15 minutes that goes by that nobody's watching that patient.”

During times of inadequate staffing, participants struggled to take adequate breaks. Although most of the participants indicated that they'd delay going to the bathroom in part because they couldn't find patient coverage, one participant rationalized her use of the bathroom without seeking coverage for her patients: “There is one thing which I'm ashamed to say, but now I will just go, I won't find someone to watch my patients, but in my head I'm like, ‘You know what, if I were going to the supply room or the medication room, I wouldn't tell anybody. I'm just going to the bathroom. I'm no further away, and I will be right back.’” This participant expressed shame about her choice to use the bathroom without patient coverage, possibly because it was contradictory to the norms of the unit. However, the participant also indicated that when they finally used the bathroom, they'd delayed voiding for a couple of hours.

Delaying voiding until they absolutely couldn't hold it any longer may be self-imposed, as evidenced by statements such as: “I just didn't want to take the time.” However, issues like inadequate staffing restricted the participants' ability to meet their biological needs to void. Additionally, some participants indicated that they'd use the bathroom “just in case” (engage in premature voiding) because they had the time available. Participants also indicated that if they used the bathroom when the urge to urinate occurred, they hurried (strained to void) because they desired to void as quickly as possible. A few participants reported feeling as though their work phones would always ring as soon as they started urinating, thus causing them to engage in straining to void: “Oh, yeah. I feel like almost every time I go to the bathroom, it's like on a cue where either a doctor calls me, or a patient always calls every time almost...Usually, I just try to...[urinate] quicker so I can answer it.”

## Discussion

The purpose of this study was to identify the toileting behaviors of nurses during the COVID-19 pandemic and the associations of different toileting behaviors with OS, SUI, and UUI. An additional aim was to qualitatively explore the stressors that nurses experienced during the pandemic that influenced bathroom behaviors at work.

Despite being healthcare professionals with functional knowledge and understanding of the benefits of healthy bladder behaviors, this sample of relatively young nurses engaged in unhealthy bladder behaviors when at work. Our sample also had a high prevalence of SUI and UUI, with 82.3% of participants experiencing some form of LUTS. This is higher than the 60% prevalence rate reported in other studies.^1^,^2^,^13^ As most women who experience SUI and/or UUI are older than 50 years of age, these results from a younger sample add valuable information to the current body of knowledge regarding nurses and LUTS development. A similar study in a population of nurses that focused on overactive bladder symptoms also found higher rates of prevalence than in the general population.^14^ This study supports and extends the notion that LUTS is an occupational hazard of nursing.

The overall mean for OS was lower than expected, considering that data collection occurred during the COVID-19 pandemic. One would think that working during the pandemic would result in a high level of perceived OS, given that many staff members had probably never worked during a pandemic because the last major pandemic was H1N1 influenza in 2009.^15^ However, many participants perceived stress, but not necessarily at an elevated level. These results could reflect that stress of the pandemic had lessened, coinciding with the delivery of our survey, which occurred after the initial wave. There may have been a decrease in the number of patients with COVID-19 and/or patient acuity on the units at the time of data collection. An additional factor could be an increase in nurses' resilience.^16^

Of the top occupational stressors reported, two were strongly related to bladder health. An inability to take an uninterrupted 30-minute mealtime break and having no time to fulfill personal needs such as drinking water and/or taking toilet breaks decrease bathroom use and increase the risk for SUI and UUI.^17^ These two specific items were also identified in the qualitative interviews, in which the participants reported that they couldn't take breaks or use the bathroom without interruption. This should be a concern for nurse managers whose nurse employees are prioritizing their job duties ahead of their health, which increases their risk for SUI and UUI and has long-term implications for their quality of life.

Surprisingly, OS wasn't associated with SUI or UUI in this study sample. However, during the interviews, participants reported that staffing levels were adequate during the initial wave of COVID-19 infection, thus increasing their ability to take bathroom breaks. During the months between when the quantitative and qualitative data were gathered, participants reported feeling an increase in stress due to nursing staff shortages. This study may have captured a shift in OS resulting from the variability in staffing during the waves of increased rates of patients with COVID-19 infection.

Premature voiding was significantly associated with SUI, which indicates that nurses feel they must take advantage of opportunities to use the bathroom when they arise. Straining to void was significantly associated with both SUI and UUI, which nurses explained in part during the interviews, reporting that they frequently attempt to hasten the voiding process in order to return to the bedside or respond to work-related phone calls. Additionally, there was a negative association between place preference and UUI, indicating that people with UUI were less likely to have place preference. Participants with UUI may use any bathroom available to avoid incontinence due to urgency.

## Implications for nurse leaders

Studies focusing on nurses have provided recommendations that could promote bladder health, such as addressing nurses' toileting behaviors and implementing organizational-level interventions such as adequate staffing.^14^,^18^,^19^ However, unless there are hospital policies in place, along with cultural shifts addressing bathroom access practices to promote bladder health, nurses' bladder health will continue to be an occupational hazard. Although OS is a part of the job, unhealthy toileting behaviors shouldn't be.

Development of policies or policy changes that prioritize breaks for nurses is the key to increasing healthy bladder habits and essential to the process of promoting bladder health. It's imperative to recognize that patient care doesn't pause when nurses are on break, especially when caring for critically ill patients. Nurse leaders should ensure that strategies are in place so that nurses don't have to worry about stepping away to use the bathroom or take a mealtime break. These strategies include increasing staffing so that someone can periodically relieve nurses during break time to ensure the delivery of patient care while nurses step away, although hiring additional nurses isn't always possible. Other strategies include creating lunch shifts and structuring breaks into the work schedule to ensure nurses get a break. Prioritizing support for breaks may help reduce the incidence of nurses denying their personal needs and prioritizing patient needs instead, which can have negative effects on bladder health. To provide true breaks for nurses, phones should be handed off, because they enable constant access that interferes with nurses' opportunities to take adequate bathroom breaks without rushing.

## Limitations

This study had several limitations. Although there were over 600 surveys submitted, we eliminated all surveys that had item nonresponses (≥5%) and those that took 5 minutes or less to complete, because submission within 5 minutes was determined to be unfeasible. Offering a drawing for one of 10 $50 gift cards could have motivated individuals to complete the survey without reading the questions before responding. Therefore, it's possible that a $50 gift card was given to an individual whose data weren't included in the analysis.

Causality among toileting behaviors, OS, and SUI and UUI couldn't be determined due to the cross-sectional design. Participants weren't asked about chronic health conditions that can affect bladder health, leading to SUI or UUI. Additionally, we didn't inquire about or exclude nurses who had a diagnosed UTI within the previous month.

## Ensuring bathroom breaks

This study highlighted important considerations regarding the bladder health of nurses caring for patients during the COVID-19 pandemic. Nurse leaders, as well as hospital administrators, need to be concerned with nurses' bladder health and should develop policies and procedures to provide adequate breaks that include patient coverage, so nurses can take uninterrupted bathroom breaks.
